# Efficient Raman Lasing and Raman–Kerr Interaction in an Integrated Silicon Carbide Platform

**DOI:** 10.1021/acsphotonics.3c01750

**Published:** 2024-02-09

**Authors:** Jingwei Li, Ruixuan Wang, Adnan A. Afridi, Yaoqin Lu, Xiaodong Shi, Wenhan Sun, Haiyan Ou, Qing Li

**Affiliations:** † Department of Electrical and Computer Engineering, Carnegie Mellon University, Pittsburgh, Pennsylvania 15213, United States; ‡ DTU Electro, Technical University of Denmark, DK-2800 KGS. Lyngby, Denmark

**Keywords:** silicon carbide, Raman scattering, Kerr microcomb, integrated photonics, nonlinear photonics, frequency comb

## Abstract

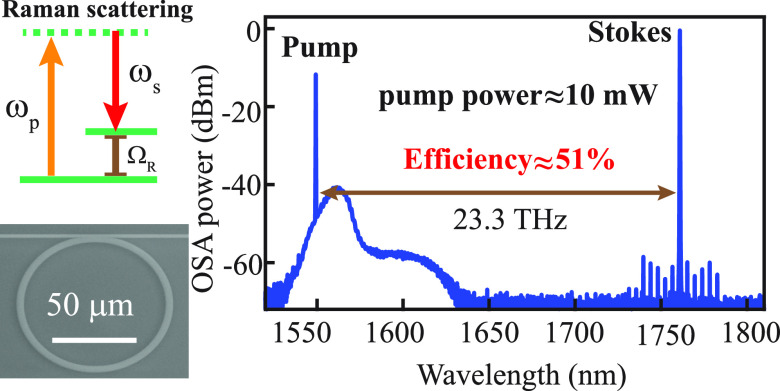

Implementing stimulated Raman scattering in a low-loss microresonator could lead to Raman lasing. Here, we report the demonstration of an efficient Raman laser with >50% power efficiency in an integrated silicon carbide platform for the first time. By fine-tuning the free spectral range (FSR) of 43 μm-radius silicon carbide microresonators, the Stokes resonance corresponding to the dominant Raman shift of 777 cm^–1^ (23.3 THz) is aligned to the center of the Raman gain spectrum, resulting in a low power threshold of 2.5 mW. The peak Raman gain coefficient is estimated to be (0.75 ± 0.15) cm/GW in the 1550 nm band, with an approximate full width at half-maximum of (120 ± 30) GHz. In addition, the microresonator is designed to exhibit normal dispersion at the pump wavelength near 1550 nm while possessing anomalous dispersion at the first Stokes near 1760 nm. At high enough input powers, a Kerr microcomb is generated by the Stokes signal acting as the secondary pump, which then mixes with the pump laser through four-wave mixing to attain a wider spectral coverage. Furthermore, cascaded Raman lasing and the occurrence of multiple Raman shifts, including 204 cm^–1^ (6.1 THz) and 266 cm^–1^ (8.0 THz) transitions, are also observed. Finally, we show that the Stokes Raman could also help broaden the spectrum in a Kerr microcomb which has anomalous dispersion at the pump wavelength. Our example of a 100 GHz-FSR microcomb has a wavelength span from 1200 to 1900 nm with 300 mW on-chip power.

## Introduction

The ubiquitous Raman effect, in which the incident photons experience inelastic scattering from the optical phonons of the matter, plays an important role in a range of applications such as material analysis,^1^ sensing,^2^ optical communication,^3−7^ and quantum information processing.^8^ By implementing the stimulated Raman scattering in a low-loss microresonator, efficient Raman lasing can be achieved when the internal Raman gain exceeds the round-trip loss, thereby extending the wavelength range of conventional laser sources. This scheme has been demonstrated in silicon and silica microresonators with sub-mW power threshold and up to 45% power efficiency.^6,9^ In the past decade, Raman lasing was also explored in wide-band-gap integrated photonic platforms such as diamond,^10^ aluminum nitride,^11^ and lithium niobate.^12,13^ While these materials exhibit well-defined Raman peaks due to their crystalline structure, the reported external power efficiency is typically less than 50% (see Table 1).

**Table 1:** Comparison of Raman Lasing in Crystalline, Wide-Band-Gap Integrated Photonic Platforms^a^

references	materials	dominant Raman shift (THz)	gain coeff. (cm/GW)	threshold (mW)	power efficiency (%)
Latawiec et al. (2015)^10^	diamond	≈40	2.5	85	<1
Liu et al.(2017)^11^	AlN	≈19	0.25–0.45	8	10–15
Yu et al. (2020)^12^	LN	≈7.5	1.3	20	≈42
**This work**	**4H-SiC**	≈**23.3**	**0.75** ± **0.15**	**2.5**	**51**

a Note that we have converted the “slope efficiency” defined in some references to the absolute power efficiency, which denotes the power ratio between the Raman signal and the pump.

**Figure 1:**
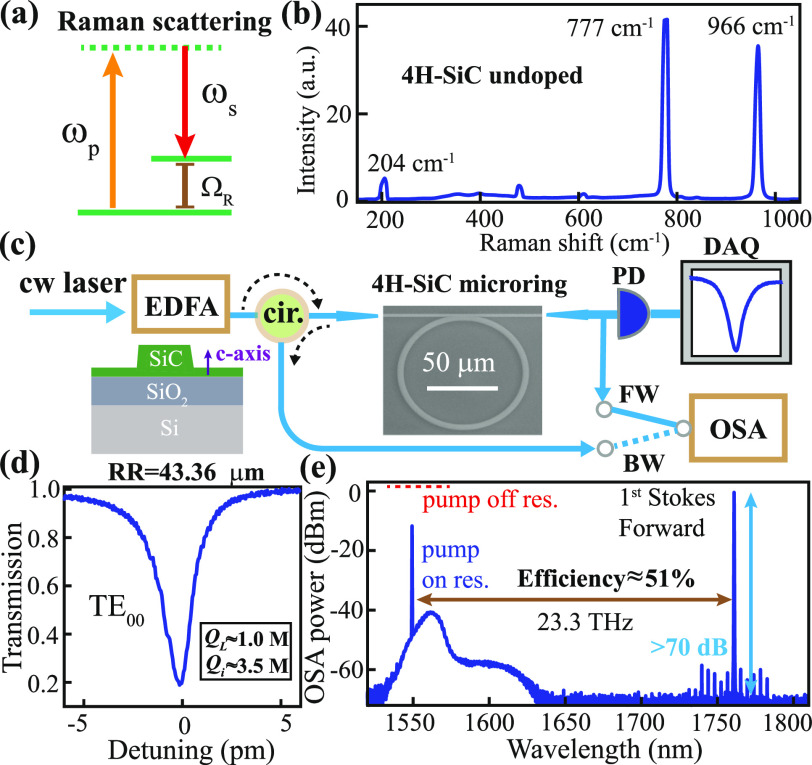
(a) Energy diagram of the Stokes Raman process: ω_*p*_, ω_*s*_, and Ω_*R*_ represent the frequencies of the pump, Stokes, and Raman shift, respectively; (b) Raman spectroscopic data of an undoped, semi-insulating 4H-silicon carbide (SiC) wafer; (c) experimental schematic: an amplified continuous-wave (CW) pump is coupled to the 4H-SiC chip through lensed fibers, whose forward (FW) and backward (BW) transmission and optical spectrum are recorded; (d) representative linear transmission of the fundamental transverse-electric (TE_00_) mode in a 43 μm-radius microring at 1550 nm; and (e) optical spectrum of the forward transmission for an approximate on-chip pump power of 10 mW (the red dashed line is the recorded power in OSA when tuning the pump laser off-resonance). EDFA: erbium-doped fiber amplifier; PD: photodetector; DAQ: data acquisition; and OSA: optical spectrum analyzer.

Recently, silicon carbide (SiC) emerged as a promising photonic and quantum material due to its unique properties, including strong Kerr nonlinearity (up to four times silicon nitride) and hosting various intrinsic and extrinsic color centers with appealing quantum properties.^14−16^ These features, combined with the demonstration of the low-loss SiC-on-insulator (SiCOI) platform,^17−19^ have resulted in a range of competitive device applications, including Kerr microcombs,^20−22^ gigahertz-level electro-optic modulators,^23^ as well as single and entangled photon sources.^24−26^ In 4H-SiC, the dominant Raman transition is around 777 cm^–1^ (Figure 1b),^27^ which in terms of the frequency shift (≈23.3 THz) is only second to that in diamond (see Table 1). To date, only Raman lasing corresponding to a frequency shift of 6.1 THz (204 cm^–1^) was reported in 4H-SiC, though the realized power efficiency was well below 1%.^19^

In this work, we report the demonstration of an efficient Raman laser in an integrated 4H-SiCOI platform for the first time. To attain a high Raman efficiency, we work with overcoupled SiC microresonators with normal dispersion around the pump wavelength (Figure 1c). To lower the power threshold, we fabricate SiC microrings with a nominal ring radius of 43 μm, which is varied by a step of 40 nm to align the Stokes resonance to the center of the Raman gain spectrum. In such compact microresonators, the fundamental transverse-electric mode has an intrinsic quality factor (*Q*) of around 3.5 million and a loaded *Q* of around 1.0 million at 1550 nm (Figure 1d). At an approximate input power of 10 mW, a strong Raman signal is observed at a frequency shift of 23.3 THz away from the pump, displaying an estimated power efficiency of 51% (Figure 1e). On further increasing the pump power, a Kerr microcomb resulting from the anomalous dispersion near the Stokes is produced, which then mixes with the pump laser through four-wave mixing to reach an even broader spectrum. A detailed study of the Raman–Kerr interaction is carried out, revealing rich physics for such a compact nonlinear system. While frequency combs have been well studied in bulky nonlinear systems such as silica fibers as well as in various integrated nonlinear platforms,^28,29^ this Raman-induced nonlinear process in a crystalline microresonator may point to an alternative appealing approach for the comb generation and spectrum expansion.^30^

## Results and Discussion

### Efficient Raman Lasing and Raman Gain Coefficient Measurement

**Figure 2:**
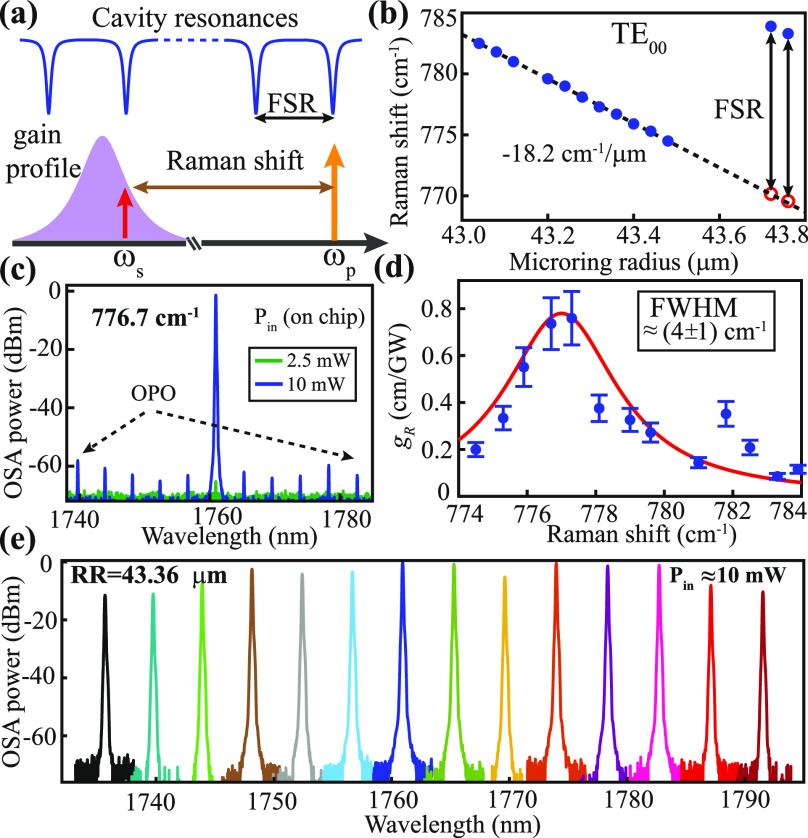
(a) Illustration of the Stokes Raman process in a microresonator. (b) Experimentally measured Raman shift (solid circles) and a linear fit (dashed line) as a function of the microring outer radius (ring width fixed at 2.5 μm). (c) Superimposed optical spectra near the 777 cm^–1^ Stokes signal at the Raman threshold power of 2.5 mW and maximum efficiency of 10 mW. The latter also results in optical parametric oscillation with the Stokes acting as the secondary pump (other spikes are the amplified spontaneous emission from EDFA transferred to the Stokes region through four-wave mixing). (d) Extracted Raman gain coefficients (markers with error bars) corresponding to different Raman shifts. The red solid line is a Gaussian fit with its full width at half-maximum (fwhm) estimated to be 120 ± 30 GHz. (e) Superimposed Stokes corresponding to the Raman shift of 776.7 cm^–1^ by varying the pump resonance in the 1529–1572 nm range for a fixed on-chip power of 10 mW.

As illustrated in Figure 2a, the effective Raman gain and frequency shift are both determined by the relative position of the Stokes resonance within the Raman gain profile. Hence, our design efforts are focused on aligning the Stokes resonance to the peak Raman gain for a low power threshold and achieving overcoupling for a high Raman efficiency. In the literature, most of the existing experiments relied upon either random frequency matching^10,11^ or using a microresonator whose FSR is smaller than the Raman gain bandwidth,^12^ both of which would result in an inflated power threshold. Here, we adopt a different approach by employing a compact microresonator (nominal radius near 43 μm with a corresponding FSR around 400 GHz) and varying its radius from 43 to 43.76 μm in 40 nm increments (20 microrings in total). A straightforward calculation predicts an incremental frequency shift of −21.7 GHz ( THz) for the 777 cm^–1^ Stokes resonance in each 40 nm increase of the ring radius. A total of 20 microrings, therefore, is able to cover the whole FSR, ensuring that there is at least one microring within 21.7 GHz of the peak Raman gain.

To obtain strong coupling at the pump and Stokes resonances, we resort to the straight coupling scheme, where a 900 nm-wide straight waveguide is evanescently coupled to the 43 μm-radius SiC microring with a 200 nm gap. The linear transmission measurement as shown in Figure 1d confirms that a coupling *Q* of 1.4 million (vs an intrinsic *Q* of 3.5 million) is attained near the pump wavelength of 1550 nm. The coupling *Q* of the 777 cm^–1^ Stokes resonance is expected to be smaller, which is typical for straight waveguide coupling due to increased modal overlap at longer wavelengths. In fact, numerical simulation points to a coupling *Q* of 0.6 million near 1760 nm (see Supplementary). This would lead to a loaded *Q* around 0.5 million of the Stokes resonance if the intrinsic *Q* is the same as 1550 nm.

With the optimized design, we proceed to fabricate the devices using a customized nanofabrication process.^20^ Briefly, we begin with a 4H-SiCOI chip with 700 nm SiC on top of 2 μm oxide (NGK Insulators, Ltd.). The pattern is first defined by spin-coating 1 μm-thick negative e-beam resist (flowable oxide, FOx-16) as the etching mask and subsequently written using a 100 kV electron-beam lithography system (Elionix ELS-G100). After development, the pattern is transferred to SiC using a CHF_3_/O_2_ plasma-etching process for an etch depth of around 575 nm, leaving an approximate 125 nm of the pedestal layer (i.e., unetched SiC). After cleaning, 2 μm oxide clad is deposited on the 4H-SiC layer to encapsulate the devices. Next, we characterize the Raman chip using the experimental schematic described in Figure 1c, where both forward and backward transmissions are measured. The fiber-to-chip coupling is achieved by implementing inverse tapers on the SiC chip, whose coupling loss is estimated to be 3–4 dB per facet (see Supporting Information for more information). The overall fiber-to-fiber insertion loss of this chip, however, is typically around 10–14 dB, as this chip suffers from relatively strong charging effects in e-beam lithography that left small unexposed areas (cracks) in random places of waveguides and microresonators (see Supporting Information for details). When appearing in waveguides, each crack introduces an approximate 2 dB additional loss. The presence of the charging-induced crack in a microring, on the other hand, will render the device useless since there is typically no resonance due to extremely low intrinsic *Q*s.

Figure 2b summarizes the measured Raman shifts for 13 microrings with different radii (the missing data points are caused by the charging effects appearing in the corresponding microrings except for the ring radius of 43.6 μm, which is discussed in Figure 4b), most of which follow the predicted linear frequency shift with the increased ring radius. The Raman shifts of the last two data points, corresponding to radii of 43.72 and 43.76 μm, are one FSR higher than the predicted trend. This can be understood as when the Stokes resonance is gradually moved toward the pump (by reducing the FSR), its adjacent resonance with a lower azimuthal order becomes closer to the center of the Raman gain and lases instead.

Among all working microresonators, the one with the 43.36 μm radius exhibits the lowest Raman threshold of 2.5 mW with a measured Raman shift of 776.7 cm^–1^ (Figure 2c). For each Raman shift, we estimate the corresponding Raman gain coefficient *g*_*R*_ based on the knowledge of the power threshold *P*_th, Raman_(3)
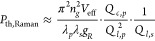
1where *n*_*g*_ is the group index (*n*_*g*_ ≈ 2.7); *V*_eff_ is the effective mode volume (*V*_eff_ ≈ 270 *μm*^3^ for 43 μm-radius SiC microrings); λ_*p*_ and λ_*s*_ denote the wavelengths of the pump and Stokes, respectively; *Q*_*c*,*p*_ and *Q*_*l*,*p*_ are the coupling *Q* and loaded *Q* of the pump resonance, respectively; and *Q*_*l*,*s*_ is the loaded *Q* of the Stokes resonance. In eq 1, the only parameter that cannot be directly measured is *Q*_*l*,*s*_, which is due to a lack of a tunable laser source near the Stokes resonance. However, as explained earlier, we can infer the coupling *Q* at the Stokes resonance based on the measured coupling *Q* at 1550 nm, while assuming that the intrinsic *Q* at the Stokes resonance is the same as the pump. Taking the pump resonance shown in Figure 1d (which corresponds to the microring with the lowest power threshold) for example, its coupling *Q* and loaded *Q* at 1760 nm are approximated to be 0.6 million and 0.5 million, respectively. These numbers can be corroborated using the fact that when the Stokes power is large enough, optical parametric oscillation (OPO) is observed near the Stokes resonance because of its anomalous dispersion (Figure 2c). (Note that the OPO pairs are generated 5 FSRs away from the Stokes while the remaining peaks observed in Figure 2c are the transferred noise from the EDFA.) The OPO threshold with the Stokes serving as the secondary pump is given by^21^
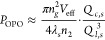
2where *n*_2_ is the Kerr nonlinear index of 4H-SiC (*n*_2_ ≈ 9.1 × 10^–19^ m^2^/W for the TE polarization).^15^ The computed power threshold of 5.0 mW is consistent with the experimental data shown in Figure 2c (the on-chip Stokes power corresponding to 10 mW input is approximately 10 × 51% = 5.1 mW). Hence, recording the OPO threshold power for each Raman shift provides an effective calibration for the estimation of the Raman gain coefficient. In Figure 2d, we plot the extracted Raman gain coefficient as a function of the corresponding Raman shift, where a Gaussian fit reveals a full width at half-maximum (fwhm) around (4 ± 1) cm^–1^ or (120 ± 30) GHz.

In addition to the efficient Raman lasing, the Stokes is expected to shift in sync with the pump resonance to maintain a fixed Raman shift. Figure 2e shows one such example for the microring with a radius of 43.36 μm, where we sequentially select pump resonances from 1529 to 1572 nm (limited by the EDFA range) for a fixed on-chip power of 10 mW and superimpose the resultant Stokes generated from 1735 to 1792 nm. The nonuniformity in the Stokes power is mainly attributed to the slight variations in the input power and resonant properties of different azimuthal orders. Nevertheless, the result displayed in Figure 2e demonstrates the ease of wavelength tuning for the Raman lasing process, which is important for many practical applications.

### Raman–Kerr Interaction and Comb Generation

**Figure 3:**
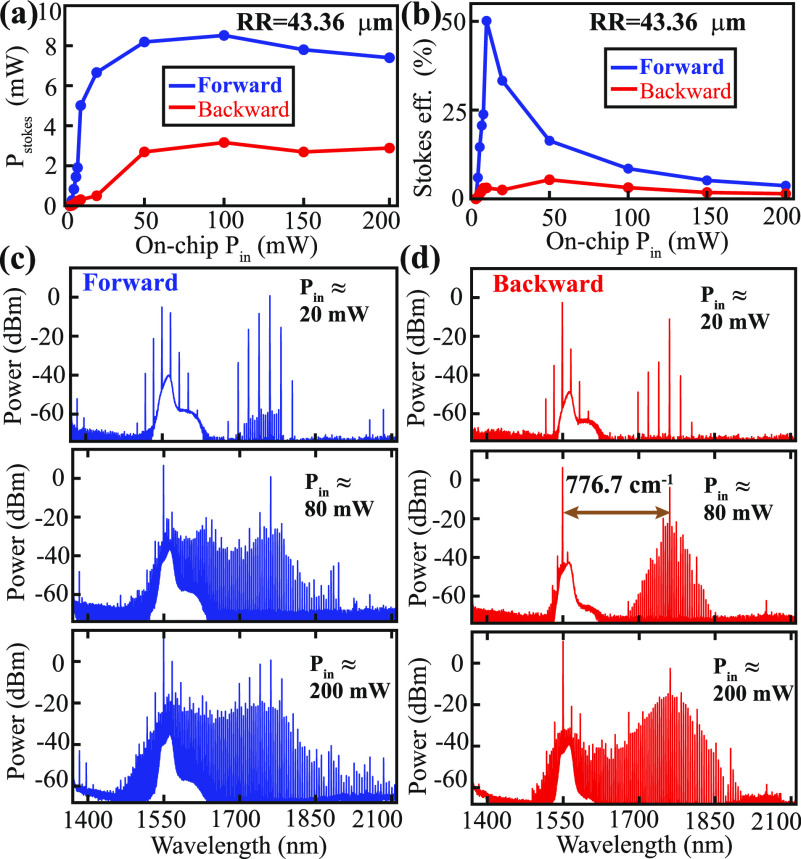
(a) Inferred on-chip Stokes power of the 776.7 cm^–1^ Raman shift for the forward and backward transmission after accounting for coupling losses; (b) Stokes efficiency by normalizing the on-chip Stokes power by the pump power; and (c,d) measured optical spectra for the forward and backward transmission at three different pump powers.

As we further increase the pump power, the Stokes signal saturates to a level of 6–8 mW on the chip for the forward transmission (Figure 3a). Hence, the maximum Stokes efficiency is achieved when the input power is around 10 mW, beyond which it begins to decrease with increased powers (Figure 3b). This is not surprising since when the Stokes power is above the OPO threshold (around 5 mW), it can act as a secondary pump for the Kerr microcomb generation due to the anomalous dispersion around the Stokes resonance (see Supplementary). In addition, the Raman process in general would result in clockwise (backward) and counterclockwise (forward) Stokes inside the microresonator,^12^ which might have slightly different power thresholds. To understand the interactive dynamics between Raman and the pump laser, we plot the optical spectra corresponding to the forward and backward transmissions at three representative powers in Figure 3c. As can be seen, a primary comb with multiple-FSR separation is first generated near the Stokes resonance at a pump power of 20 mW (which can be compared to Figure 2c to support the OPO identification). These comb lines are also transferred to the pump region due to a nondegenerate four-wave mixing process (so-called four-wave mixing Bragg scattering): ω_comb near pump_ = ω_comb near Stokes_ + ω_*p*_ – ω_*s*_.^31^ In fact, the small peaks near the Stokes resonance are the transferred noise from the EDFA (amplified spontaneous emission) based on the same principle: ω_noise near Stokes_ = ω_noise near pump_ + ω_*s*_ – ω_*p*_. The backward spectrum at 20 mW consists of the reflection of the pump and the backward-propagating Stokes comb generated inside the microring. As we increase the pump power to 80 mW, we begin to see major differences in the forward and backward optical spectra: the backward spectrum contains a filled Kerr microcomb (termed as the Raman–Kerr comb for simplicity) that is well separated from the 1550 nm pump, while the forward spectrum consists of additional comb lines between the pump and the Stokes resonance. Such difference can be understood as the forward optical spectrum consists of the counterclockwise Raman–Kerr comb and the pump laser, both of which propagate in the same direction inside the microresonator and hence interact with each other through four-wave mixing. In contrast, the backward spectrum consists of the reflected portion of the pump and the clockwise Raman–Kerr comb without mixing with each other inside the microresonator. Finally, as the pump power is increased to 200 mW, both forward and backward spectra are significantly widened, though the forward spectral coverage is modestly wider, spanning from 1400 to 2100 nm.

**Figure 4:**
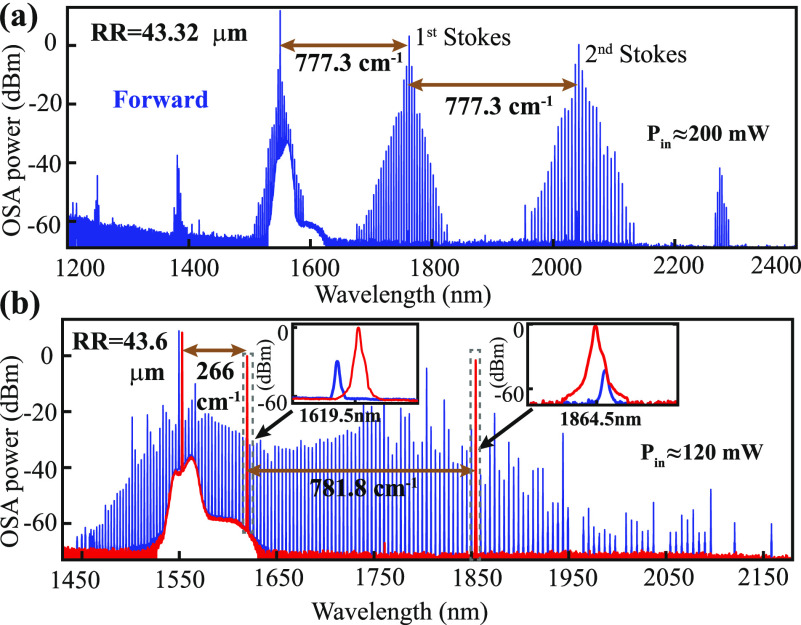
(a) Cascaded Raman shifts of 777.3 cm^–1^ from the microring with an outer radius of 43.32 μm at an input power of 200 mW. (b) The blue solid line is the Raman–Kerr comb from the microring with an outer radius of 43.6 μm when pumped at the TE_00_ resonance near 1549.5 nm. The red solid line corresponds to pumping at the next resonance near 1552.9 nm, whose spectrum consists of two cascaded Raman shifts, one near 266 cm^–1^ and the other near 781.8 cm^–1^, without the Kerr comb. The insets show the zoom-in spectra near the two Raman shifts, suggesting that the Stokes is likely formed by a different mode family (TM).

In addition to the Stokes-induced Kerr microcomb, we also observe cascaded Raman generation in a few microrings, pointing to even more complicated interactions between the Raman and Kerr effects.^5,19,32^ For example, Figure 4a shows that there are two cascaded 777.3 cm^–1^ Stokes in the microring with a radius of 43.32 μm, each generating a Kerr microcomb in the adjacent region. Moreover, while the 777 cm^–1^ Stokes dominates over other Raman transitions in most of the microring resonators, we also observe different Raman shifts in certain geometries. For example, in the microring with a radius of 43.6 μm, when pumping the TE_00_ resonance near 1549.5 nm, a regular Raman–Kerr comb is observed in the forward transmission (blue solid line in Figure 4b). However, when we tune the pump laser to the 1552.9 nm resonance, only two cascaded Raman shifts are observed in the spectrum, one corresponding to 266 cm^–1^ and the other corresponding to 781.8 cm^–1^ (red solid line in Figure 4b). An inspection of the optical spectrum near the two Stokes (see the two insets in Figure 4b) suggests that the two Stokes are not from the TE_00_ mode family. Instead, they are likely the TM_00_ modes of the microring that happen to be accidentally frequency-matched to the TE_00_ pump for the observed Raman transitions. This may explain why the 266 cm^–1^ Raman transition is often absent in the bulk-wafer measurement (see Figure 1b).^27^

**Figure 5:**
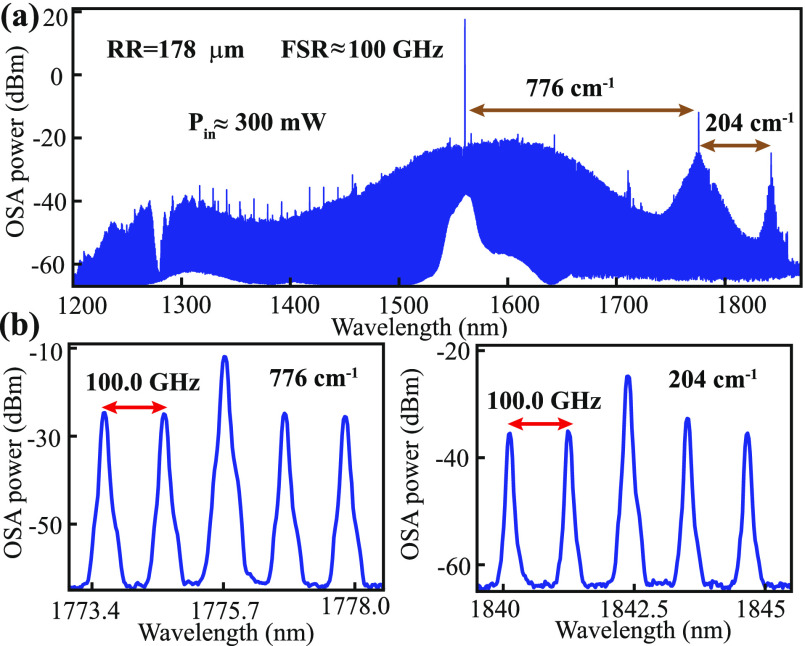
(a) Optical spectrum of a SiC microring with a radius of 178 μm (corresponding free spectral range around 100 GHz), which consists of the Kerr comb from the pump itself and two cascaded Raman peaks near 776 and 204 cm^–1^. (b) Zoomed-in spectra near the two Raman peaks.

So far, we have only discussed the stimulated Raman scattering in SiC microresonators that exhibit normal dispersion at the pump wavelength, a preferred configuration to avoid competition between the Raman and Kerr effects at the same wavelength.^33^ It is possible, however, to observe Stokes appearing in a Kerr microcomb as well. Figure 5 shows one such example for a 178 μm-radius SiC microring resonator. The TE_00_ mode at this increased radius possesses a weak anomalous dispersion (see the Supporting Information for more details). When pumped at 300 mW power, a broad Kerr microcomb spanning from 1200 nm to 1900 nm is generated along with two cascaded Raman transitions, with one peak corresponding to 776 cm^–1^ and the other to 204 cm^–1^. A zoomed-in view of the two Raman peaks reveals that they are from the same mode family.

Finally, we want to comment on the coherence properties of the Raman–Kerr comb generated in this work. Most of them, with the only exception of the primary comb in Figure 3c, are believed to be in a chaotic state that is not phase-locked to the pump laser. It is possible, however, to observe a soliton microcomb with the Stokes serving as the secondary pump or the coexistence of the Stokes soliton with the Kerr soliton generated by the pump.^30,33^ A detailed discussion on these aspects of Raman–Kerr interactions is beyond the scope of this paper and will be left to future works.

In conclusion, we performed a thorough investigation of the Raman effect in low-loss 4H-SiC microresonators, resulting in the demonstration of an efficient Raman laser with >50% power efficiency and a detailed characterization of the Raman gain coefficient for the dominant 777 cm^–1^ (23.3 THz) Stokes transition, both of which are the first in an integrated SiC platform (to the best of our knowledge). In addition, our study of the Stokes-induced Kerr comb and its interplay with the pump laser revealed the different roles of four-wave mixing in the forward and backward transmissions as well as the rich interactions between the Raman and Kerr effects. Finally, we also observed the occurrence of other Raman transitions such as 204 cm^–1^ (6.1 THz) and 266 cm^–1^ (8.0 THz) along with the 777 cm^–1^ Stokes, a feature that can be utilized to broaden the Stokes-induced or pump-induced Kerr microcombs. We believe that our results fill up the gap in the understanding of the stimulated Raman process in 4H-SiC microresonators, which could potentially lead to a myriad of applications including efficient Raman lasers and novel approaches to generate broadband microcombs.

## Data Availability

Data underlying the results presented in this paper are not publicly available at this time but may be obtained from the authors upon reasonable request.

## Supporting Information Available

Additional discussions on the inverse tapers, charging effect, dispersion of 43 μm-radius SiC microrings, waveguide-resonator coupling, and 178 μm-radius SiC microrings (PDF)

## Notes

Associated Content: Jingwei Li, Ruixuan Wang, Adnan A. Afridi, Yaoqin Lu, Xiaodong Shi, Wenhan Sun, Haiyan Ou, and Qing Li, “Efficient Raman lasing and Raman–Kerr interaction in an integrated silicon carbide platform”, 2023, arXiv: 2311.06561.
