# Supplementary material for: Efficient Raman Lasing and Raman–Kerr Interaction in an Integrated Silicon Carbide Platform
Source: ACS Photonics. 2024 Feb 9;11(2):795–800. doi: 10.1021/acsphotonics.3c01750 (PMC10885207; doi:10.1021/acsphotonics.3c01750)
Supplement: Supplementary file 1 — ph3c01750_si_001.pdf [file ph3c01750_si_001.pdf]

Supporting Information: Efficient Raman lasing and Raman-Kerr interaction in an integrated silicon carbide platform

JINGWEI LI[1+], RUIXUAN WANG[1+], ADNAN A. AFRIDI[2], YAOQIN LU[2], XIAODONG SHI[2], WENHAN SUN[1], HAIYAN OU[2], AND QING LI[1]

[1] *Department of Electrical and Computer Engineering, Carnegie Mellon University, Pittsburgh, PA 15213, USA*
[2] *DTU Electro, Technical University of Denmark, DK-2800 KGS. Lyngby, Denmark*
[+] *These authors contributed equally to this work.*
[*] *qingli2@andrew.cmu.edu*

Additional discussions on the inverse tapers, charging effect, dispersion of 43-μm-radius SiC microrings, waveguide-resonator coupling, and the 178-μm-radius SiC microring. Contains 5 pages and 5 figures.

1. Inverse tapers and charging effect

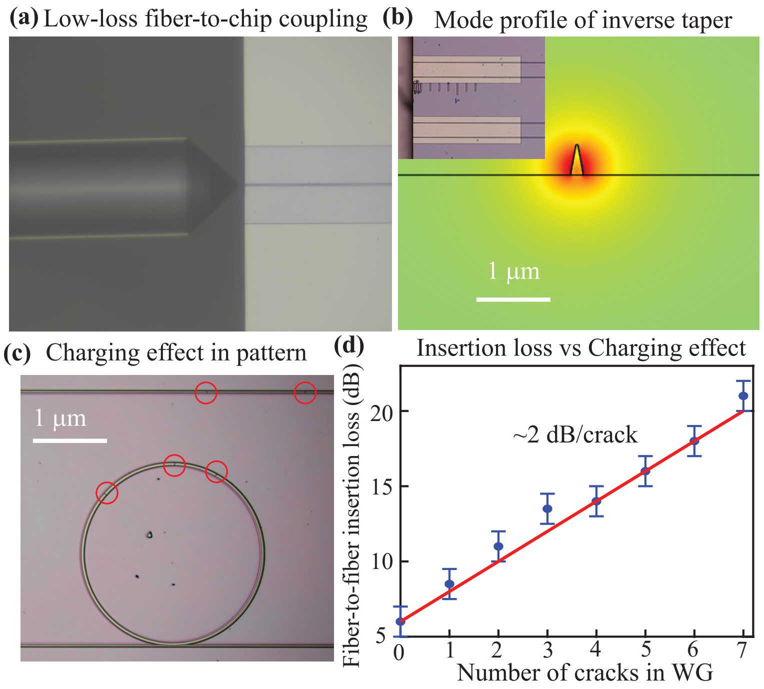

Fig. S1. (a) Optical micrograph of a lensed fiber aligned to a polished SiC facet; (b) The mode profile of the SiC inverse taper with a (bottom) width around 250 nm. The inset shows the optical micrograph of the taper region where the pedestal layer is removed by an additional step of lithography; (c) Optical micrograph highlighting charging-induced cracks (red circles) in waveguides and microresonators; and (d) Statistically averaged fiber-to-fiber insertion loss as a function of the number of cracks appearing in waveguides.

In this work, efficient fiber-to-chip coupling is achieved by aligning a lensed fiber (with a mode field diameter of 2.5 μm) to the SiC inverse taper implemented at the chip facet (Fig. S1(a)). The mode profile of the inverse taper is provided in Fig. S1(b), which has an estimated coupling loss of 2 dB by computing its modal overlap with the lensed fiber. Given that the presence of a pedestal layer hinders the mode expansion and thus reduces the coupling efficiency, we remove the pedestal layer in the taper region using an additional step of lithography (see the inset of Fig. S1(b)). That is to say, in the microring region the SiC is 700 nm thick with 125 nm pedestal, while the SiC is only around 550 nm thick without pedestal in the taper region. We then polish the SiC facets so that the tip of the inverse tapers is only a few microns away from the facet. In our prior experiments, the inverse tapers would typically fail at high optical input powers due to residues from the polishing step that caused strong optical absorption [1]. After switching to a more thorough cleaning process, the inverse tapers can now sustain optical powers up to several watts without getting burnt.

When writing long waveguides in the e-beam lithography, the charging effect caused by charge accumulation in insulating substrates becomes more apparent, resulting in cracks in the writing area as highlighted in Fig. 1S(c). Though a few mitigating methods including evaporating a thin layer of aluminum on top of the e-beam resist have been tried, it is still common to get 1-3 cracks in a 5-mm-long waveguide. For dense patterns consisting of many devices (including this

Raman chip), the charging effect can be even stronger. Fitting the measured insertion loss as a function of the observed cracks in various waveguides reveals a positive correlation between the increased losses and the number of charging-induced cracks. Statistically, each crack introduces an approximate 1.5-2 dB additional loss. For waveguides that are free of the charging effect, the total chip insertion loss is only around 6-7 dB, suggesting a coupling loss around 3 dB for each inverse taper (which is slightly higher than the 2 dB number obtained from simulation).

2. Dispersion of 43-μm-radius SiC microrings

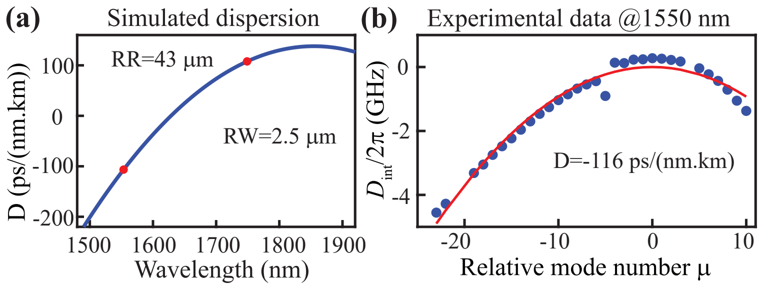

Fig. S2. (a) Simulated dispersion of the 43-μm-radius SiC microring used in this work: a ring width of 2.5 μm exhibits normal dispersion around 1550 nm and anomalous dispersion around 1760 nm (as highlighted by the two red dots in the figure); and (b) Experimentally measured dispersion by computing the integrated dispersion D_{int} as a function of different azimuthal orders, confirming normal dispersion in the 1550 nm band. D_{int} is defined as $D_{\text{int}} \equiv \omega_\mu - \omega_0 - D_1\mu$ where μ is the relative azimuthal order to the pump resonance (i.e., $\mu = 0$ for the pump mode), ω_μ is the corresponding resonance frequency, and D_1 is the free spectral range of the resonator.

As discussed in the main text, the 43-μm-radius SiC microring employed in this work is designed to exhibit normal dispersion near the pump wavelength (1550 nm) while possessing anomalous dispersion in the Stokes wavelength corresponding to the dominant 777 cm^{-1} Raman shift (1760 nm). Numerical simulation performed in Fig. S2(a) suggests that this can be achieved by choosing a ring width of 2.5 μm with a height of 700 nm and a pedestal layer of 125 nm. After fabricating these microrings, a linear dispersion measurement was performed to confirm the normal dispersion in the 1550 nm region (see one example in Fig. S2(b)).

3. Waveguide-resonator coupling simulation

The straight coupling scheme, as illustrated in Fig. S3(a), is ideal for achieving over-coupling in both the pump and Stokes resonances. This is because in this coupling scheme, the modal overlap factor plays a dominant role (given the short interaction length), which typically results in stronger coupling at longer wavelengths [2]. This means if we can achieve over-coupling in the 1550 nm band, the degree of over-coupling at 1760 nm should be even stronger (see Fig. S2(b) for simulation). Typically, due to the limited accuracy in the dimensional control from nanofabrication, we have to adjust the gap by up to 50 nm to match the observed coupling Q with the experimental data. In this case, there is good agreement between the measured coupling Q at 1550 nm and the simulation data based on the designed gap (200 nm). Nevertheless, once we match the coupling Q at the pump wavelength (1.4 million at 1550 nm), numerical simulation predicts an approximate coupling Q of 0.6 million at 1760 nm. This number is also verified by comparing the predicted OPO power threshold with the experimental data (see main text).

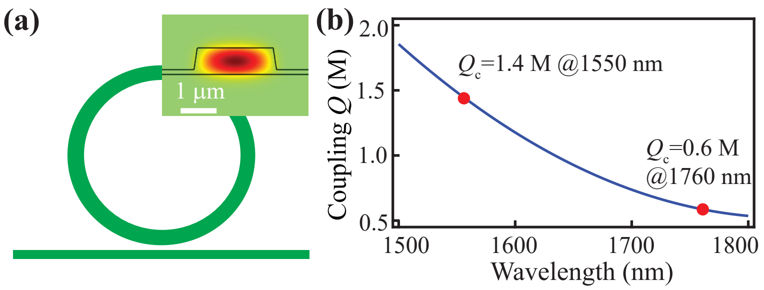

Fig. S3. (a) Schematic of straight waveguide coupling between a microring resonator and the access waveguide. The inset shows the mode profile of the 43-μm-radius SiC microring used in this work, which has a width of 2.5 μm and a height of 700 nm (the pedestal layer is 125 nm). (b) Simulated coupling Q as a function of wavelengths: the access waveguide has a width of 900 nm and a coupling gap of 200 nm.

4. Raman power efficiency estimation

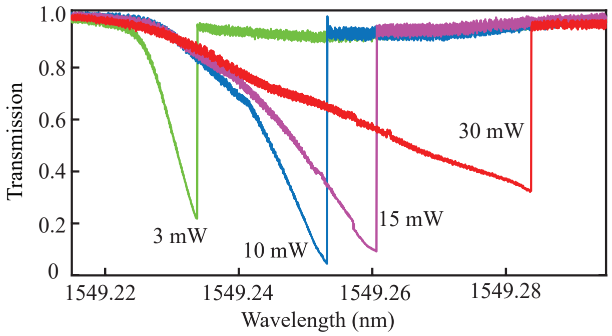

Fig. S4. Normalized pump transmission of the 43.36-μm-radius SiC microring at different input powers.

The Raman power efficiency around 51 % is estimated based on the OSA spectrum shown in Fig. 1(e), which is given by the ratio between the recorded Stokes power (when the pump is tuned into resonance) and the off-resonance pump power. This measurement assumes that the Stokes and pump experience the same out-coupling loss from the SiC chip (the numerical value of the insertion loss is less important as long as it stays the same between the two measurements). It is possible, however, that the coupling loss of the 1760 nm Stokes is slightly higher than that of the 1550 nm pump, as suggested by preliminary characterization carried out by the DTU team using a supercontinuum source on a different SiC chip. This would indicate a slightly higher power efficiency (by 0.5 dB) if verified. To be conservative, the reported number here does not adjust for the difference of the insertion loss between the Stokes and pump.

One may notice that the extinction ratio of the pump resonance shown in Fig. 1(e) ($\approx$ 14 dB) is higher than what is suggested by the linear transmission in Fig. 1(d) ($\approx$ 7 dB). This is confirmed by comparing the pump transmissions at various input powers in Fig. S4. The result is also consistent with the interpretation of the over-coupled pump resonance in the linear regime and high Raman conversion efficiency that depletes the pump when the input power is around 10 mW.

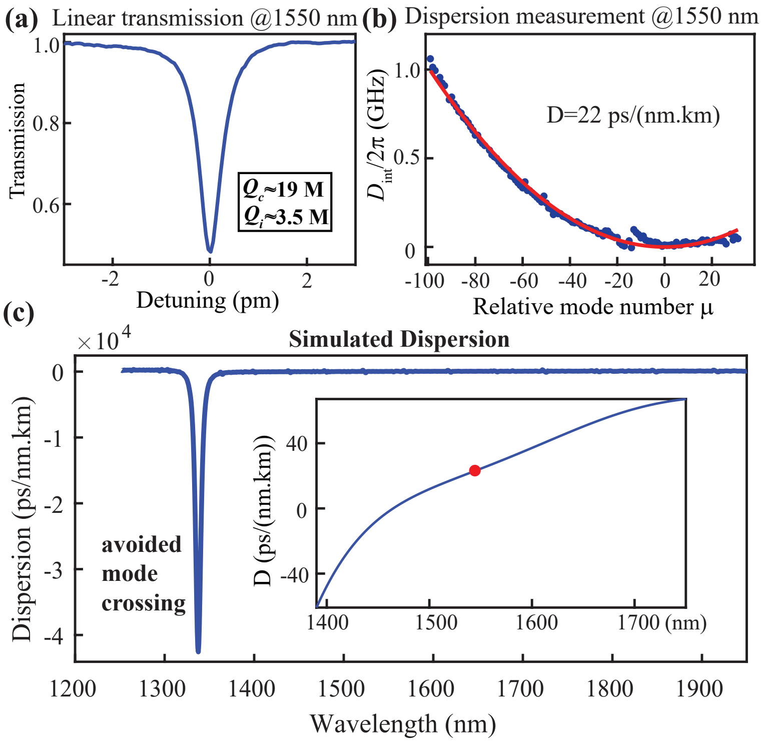

Fig. S5. (a) Representative linear transmission of the fundamental TE_{00} mode of the 178-μm-radius SiC microring studied in Fig. 5 of the main text, showing an intrinsic Q around 3.5 million and a coupling Q around 19 million. The microring has a ring width of 3.5 μm and an access waveguide with a width of 1450 nm and a pulley coupling length of 30 μm; (b) Dispersion measurement confirms anomalous dispersion in the 1550 nm band, which is also consistent with simulation shown in (c) at 1550 nm; and (c) Simulated dispersion for the TE_{00} mode of the 178-μm-radius SiC microring, which shows an avoided mode crossing around 1330 nm.

5. Additional information on 178-μm-radius SiC microrings

The 178-μm-radius SiC microring is from a different chip which was fabricated using a similar process but with different designs. Notably, it has the same SiC thickness of 700 nm and a pedestal layer of 125 nm. The most significant difference is that the coupling to the fundamental TE_{00} mode is based on the pulley coupling instead of straight coupling [2]. The linear transmission shown in Fig. S5(a) reveals that the TE_{00} mode is under-coupled at 1550 nm, which explains why the Raman signals are much weaker compared to those observed in 43-μm-radius SiC microrings. The dispersion characterization in Fig. S5(b) confirms that the larger-radius-microring exhibits weak but anomalous dispersion near 1550 nm, which is consistent with the simulated dispersion (see Fig. S5(c)). For the 700 nm SiC with 125 nm pedestal, numerical simulation also points to an avoided mode crossing between the TE_{00} and TM_{00} mode families around the wavelength of 1300 nm. This may explain the disappearance of certain comb lines in that region as shown in Fig. 5(a) in the main text.

References

1. L. Cai, J. Li, R. Wang, and Q. Li, "Octave-spanning microcomb generation in 4H-silicon-carbide-on-insulator photonics platform," Photonics Res. **10**, 870–876 (2022).

2. Q. Li, M. Davanço, and K. Srinivasan, “Efficient and low-noise single-photon-level frequency conversion interfaces using silicon nanophotonics,” Nat. Photonics **10**, 406–414 (2016).
